# Papilloma of the Bladder

**Published:** 1909-06-26

**Authors:** 


### PAPILLOMA OF THE BLADDER.

Villous growths of the bladder, although they are innocent as far as their microscopical characteristics are concerned, are particularly malignant in this respect, that they tend sooner or later to cause the death of their possessor.

Histologically a vesical papilloma is seen to consist of a large number of delicate processes covered with the normal transitional epithelium of the bladder, but none of these invade the basement membrane and run riot in the subepithelial tissue as in a typical carcinoma; and it must therefore be classified as a non-malignant growth. But its rapid growth and its power of infecting the opposite bladder wall wherever it comes into contact with it, and the fact that it eventually kills the patient, render it, clinically speaking, highly malignant.

The first symptom to which it gives rise is nearly always an attack of hæmaturia, which is generally slight, and passes off in a day or two, so that unfortunately the patient does not think it worth while to seek medical advice. The hæmaturia is repeated at intervals, but on each occasion the amount of blood lost becomes more and the intervals of freedom become shorter, until a time comes when the hæmorrhage is so profuse that the patient may become absolutely blanched. Intermittent hæmaturia of the character described is almost pathognomonic of vesical papilloma.

It is a little difficult to account for the intermittent nature of the hæmorrhage, but the most feasible explanation is that as the papilloma grows its processes, each of which carries in it one or more delicate vessels, are subjected to pressure by the contractions of the bladder at the end of micturition, and their tips are broken off. Slight bleeding ensues, which stops spontaneously; the papillomatous processes begin to grow again, and when they have reached a certain size the same thing happens again. But the growth is also expanding at its base, and becoming larger, so that it occupies a greater portion of the bladder. Thus it happens that the bleeding is more frequent, and with each regeneration the processes themselves become larger, so that more blood is lost, and in the later stages the amount may be positively alarming.

As the disease progresses other symptoms make their appearance, the most prominent of which is increased frequency of micturition, both by day and night. In the later stages the condition is also associated with pain.

### Methods of Diagnosis.

It follows from what has been said that it is of the utmost importance to diagnose the condition in the early stages, in which operation holds out the prospect of a radical relief.

A careful examination of the urine, if it is examined during one of the attacks of hæmaturia, should reveal sufficient evidence to arouse suspicion as to the true nature of the case. If the urine is centrifugalised and examined microscopically it is generally possible to find small fragments of papilloma in the sediment.

No time should then be lost in examining the interior of the bladder with the cystoscope. It is possible to do this without giving the patient an anæsthetic, but it is not advisable. Of course, in an out-patient department, where a number of such examinations have to be made in one afternoon, there is not sufficient time to give all the patients an anæsthetic. In this case the urethra should be made insensitive before passing the instrument by injecting a 10 per cent. solution of cocaine. But to render this effectual the cocaine must be massaged well down into the posterior urethra. It is not sufficient simply to inject it into the external urinary meatus. But, as has been said, it is advisable, whenever possible, to give an anæsthetic.

In order to get a clear view of the interior of the bladder it is essential to irrigate it thoroughly before cystoscopy with some non-irritant fluid, preferably saline or boracic solution, through a catheter, and the irrigation must be continued until the fluid returns quite clear, otherwise a satisfactory view cannot be obtained. The great difficulty in cases in which the disease is at all advanced is that the passage of the cystoscope is in itself sufficient to start the hæmorrhage again, and it is then impossible to get a satisfactory view. But where one is dealing with a single papilloma it can be seen hanging from the bladder wall, rather like a sea anemone as it lies under the surface of the water.

### Operative Treatment.

As soon as the diagnosis is established no time should be lost in performing a suprapubic cystotomy and removing the growth. A few practical points in the operation are worthy of mention. After opening the bladder, it is well to pass a couple of sutures through the cut edge on either side, and tie them to the edges of the wound. This is only a temporary measure. At the end of the operation the stitches can be removed. They are very serviceable, because they hold the bladder well up into the wound, and they avoid the necessity of putting clips on the bladder wall. The interior of the bladder is next examined. This is best done through a large speculum, like a Ferguson's vaginal speculum, only that its end is at right angles to its long axis. A head lamp is necessary. By putting the speculum down on to the bladder wall the position and extent of the tumour can be estimated.

The ideal operation is to remove the tumour entire with a pair of curved scissors, taking care not to go through the bladder wall. Hæmorrhage often follows, and if this cannot be controlled by irrigation with hot saline and adrenalin, the bleeding points must be looked for through the speculum and clamped. The clamp may be left *in situ* for a day or two. It is better to do this than to attempt to ligature it, since the presence of a ligature in the bladder acts as a basis for a phosphatic calculus. When the tumours are large and multiple, with broad bases, complete eradication is impossible, and it will generally be found that only the most prominent portions can be got away.

